# Perceived Noise Pollution and Self-Reported Health Status among Adult Population of Bangladesh

**DOI:** 10.3390/ijerph19042394

**Published:** 2022-02-19

**Authors:** Md. Mostafizur Rahman, Farah Tasnim, Masrur Abdul Quader, Md. Nafee-Ul-Islam Bhuiyan, Mohammed Sadman Sakib, Rawnok Tabassum, Ifta Alam Shobuj, Lamia Hasan, Musabber Ali Chisty, Farzana Rahman, Edris Alam, Abu Reza Md. Towfiqul Islam

**Affiliations:** 1 Department of Disaster and Human Security Management, Faculty of Arts and Social Sciences, Bangladesh University of Professionals, Mirpur Cantonment, Dhaka 1216, Bangladesh; mostafizur@bup.edu.bd (M.M.R.); farah.tasnim@bup.edu.bd (F.T.); maq9811@gmail.com (M.A.Q.); nafee.bhuiyan.08@gmail.com (M.N.-U.-I.B.); sakibmhs@gmail.com (M.S.S.); moumee57@gmail.com (R.T.); iftealamshobuj@gmail.com (I.A.S.); lamiahasan122@gmail.com (L.H.); 2 Institute of Disaster Management and Vulnerability Studies, University of Dhaka, Dhaka 1000, Bangladesh; musabber.chisty@du.ac.bd; 3 Department of Computer Science and Engineering, Independent University, Dhaka 1212, Bangladesh; farzana.rahman@iub.edu.bd; 4 Faculty of Resilience, Rabdan Academy, Abu Dhabi 22401, United Arab Emirates; ealam@ra.ac.ae; 5 Department of Geography and Environmental Studies, University of Chittagong, Chittagong 4331, Bangladesh; 6 Department of Disaster Management, Begum Rokeya University, Rangpur 5400, Bangladesh

**Keywords:** noise exposure, noise perception, self-reported health status, Bangladesh

## Abstract

Despite the public health concern, there is a dearth of research regarding perceived noise pollution and noise-related health status in Bangladesh. This study was carried out to evaluate the noise-related health status among Bangladesh’s adult population. 1386 adult Bangladeshis participated in an online survey. A linear regression model was used to evaluate overall noise-related health status determinants. 91% of the survey population reported noisy environments in their neighborhood, with the majority reporting two types (34%) of noise pollution sources. Road vehicles (38%) and construction activities (24%) were identified as significant source of noise pollution. The Bangladeshis are primarily exposed to noise during school and office hours. Socio-demographic information, perceived noise pollution and individual views towards noise pollution were examined as determinants of noise-related health problems. Females were found to be more impacted than males, and young people also expressed concern about noise pollution’s influence. Residents in mixed-unit buildings exhibited a significant level of noise-related health problems such as deafness, insomnia, heart disease, headache, stress, poor concentration, production loss, fatigue, irritability, heartburn, indigestion, ulcers, and high blood pressure. Noise pollution from road vehicles and industry has been shown to have a negative effect on people’s health. Individuals affected by noise were interested in noise reduction efforts. The findings of this research may aid in the improvement of international, national, and local noise control efforts.

## 1. Introduction

Noise pollution has developed into a significant public health problem [1,2,3]. Numerous studies have examined the detrimental effect of noise pollution on people’s health [1,4,5,6,7]. The burden of noise pollution’s health consequences is estimated to be the second highest after air pollution [3,8]. It has been recognized as a significant environmental stressor associated with various illnesses such as deafness, insomnia, heart disease, headache, stress, poor concentration, production loss, fatigue, irritability, heartburn, indigestion, ulcers, and high blood pressure [5]. Over time, prolonged exposure to excessive noise (over 70 dB(A)) can have a detrimental effect on physical health and psychological well-being [3,9]. 

The way people perceive noise pollution affects their mobility decisions, perceived health status, sense of well-being, and general quality of life [2,10]. Numerous studies have demonstrated the significance of subjective and objective noise pollution [2,10,11,12]. Noise pollution could be assessed by using observation instruments, and through individuals’ perception level. Perceived noise pollution can be used in conjunction with observed noise pollution [2]. Nonetheless, perceived noise pollution substantially affects human health [10,12,13]. It is critical to analyze perceived noise pollution in order to enhance residents’ perceptions of their health, overall well-being, and contentment [2].

Although residents’ perceptions of noise were consistent with documented noise levels (stationary measurements/maps), residents’ discomfort is rarely addressed [11,12,14]. Self-reported health represents an individual’s psychological, social, and physical health that can’t be quantified by a single measure of morbidity [13,15]. Because self-reported health is inextricably linked to this broader picture of health, low self-reported health is highly associated with premature mortality [15]. However, self-reported health surveys may contain a range of health statuses caused by various social, economic, and environmental factors. For instance, in the majority of situations, self-reported health questionnaires consist of a single straightforward question (How would you rank your general health?) followed by three to five ratings (poor, medium, fair health condition). Socio-economic status and environmental problems such as air pollution, noise pollution, and waste pollution may contribute to health status [2]. 

Noise pollution has emerged as a significant health hazard in Bangladesh [16,17,18,19]. Bangladesh’s Department of Environment found that noise levels in Bangladesh, particularly in large cities, are significantly over what is considered a tolerable threshold of noise for humans. In a 2017 survey, they revealed that around 12% of the population in Bangladesh had lost their hearing due to noise pollution [16]. Bangladesh’s Department of Environment issues several laws and guidelines. However, they require proper review and execution [16]. Vulnerable regions and persons must be recognized objectively and subjectively. The purpose of this study was to examine perceived noise pollution and its connection with self-reported health status among adult Bangladeshi populations. We considered several noise-related health issues highlighted in previous studies when assessing self-reported health status [4,5,10]. Several studies on noise pollution in Bangladesh have previously been performed [18,19,20]. However, to our knowledge, this is the first study to examine perceived noise pollution and self-reported noise-related health problems among Bangladesh’s adult population. The outcomes of this study may aid in the development of national and local noise pollution reduction strategies. 

## 2. Materials and Methods

### 2.1. Study Design and Ethical Issue

The current cross-sectional study employed an online survey to collect data from adults in Bangladesh. A self-reported health survey was designed to ascertain respondents’ perceptions of noise-related health problems. This study was conducted as part of a research project (Ref. DHSM-2021/2) approved by the Disaster and Human Security Management department of Bangladesh University of Professionals in Dhaka, Bangladesh. It has maintained all associated ethical issues following human subjects. Online consent was obtained from individuals who participated anonymously. The questionnaire’s cover page clearly stated that the data would be utilized solely for research purposes. No incentives were granted for participation in the survey. 

### 2.2. Survey Tool 

The draft questionnaire was developed using previous research and discussion with experts [1,3,4,5,6,18,19,20,21]. Additionally, a pilot survey was conducted among a sample of university students to validate the questionnaire. The final questionnaire (local Bangla and English version) consisted of six sections: socio-demographic information, perceived extreme noise pollution time within the locality, source of noise-related information (available media for noise pollution related information), perceived noise pollution in the locality, individual’s views concerning noise pollution, and the noise-related health problem. The perceived extreme noise pollution time section includes a straightforward question such as “When is the noise pollution at its worst in your locality?”, whereas the most frequently used source for noise-related information included a question such as “Which media do you use the most for information about noise pollution and precautionary measures?”. Additionally, the questionnaire included questions concerning the type of noise pollution source, the major source of noise pollution, the noisiest time of day or night, and the status of noise pollution during the COVID-19 lockdown period in order to ascertain an individual’s perception of noise pollution in their locality. We included items to ascertain respondents’ views towards noise pollution (discussions about noise pollution with neighbors, sensitivity to noise pollution, whether they repaired their home to lessen noise pollution, whether they are willing to pay annually to lessen noise pollution, and whether they prioritize a noise-free living place even with high-cost). The final section consisted of one question, “Do you suffer from any of the following?” regarding their noise-related health problem. It included 13 noise-related health problems, such as deafness, insomnia, heart disease, headache, stress, poor concentration, production loss, fatigue, irritability, heartburn, indigestion, ulcers, and high blood pressure [5] with “Yes/Maybe/No/I don’t know” option. None of the participants responded as “I don’t know” in this section. Positive responses received a score of 1, while neutral and negative responses received scores of 0.50 and 0, respectively. 

### 2.3. Data Collection and Sampling

The online survey took place between April and June 2021. Google Forms was used to create the questionnaire. A group of university students was recruited (based on their research experience) to distribute the questionnaire via Facebook, Google Classroom, WhatsApp, and email to the people in Bangladesh. Boundary conditions were age (18 years and above), living in Bangladesh during the survey, and internet access. This study followed a non-probability sampling technique. According to Morgan’s Table, a minimum sample size of 384 individuals (95% Confidence Interval) was required for this perception-based study [22]. We approached an overwhelmingly high number of potential respondents, around 2600 individuals. We obtained responses from 2290 individuals (88.08% of the approached potential responders). However, we included only the individuals who had lived in their current location for at least five years. Finally, the analysis took into account the responses of 1386 participants. 

### 2.4. Data Analysis

Python (version 2.7; Beaverton, OR 97008, USA) and RStudio (version 1.2.5042; Boston, MA, USA) were used for data management and statistical analyses [23,24]. Descriptive statistics (frequency and percentage) were calculated when applicable. The sum of the 13 noise-related health problems scores was used to determine the overall score for noise-related health problems. The overall noise-related health problems could indicate the individual’s health problems due to the noise pollution. A linear regression analysis was conducted to examine the determinants of self-reported overall noise-related health problems. In all statistical analyses the 95% Confidence Interval (CI) was applied. 

## 3. Results

### 3.1. Socio-Demographic Profile

Table 1 presents the overall noise-related health problems of the respondent shown in relation to their socio-demographic profiles. This study had younger participants, 56% female, and 44% male ones. The majority of them were unmarried, living with their family members. Many individuals were living in Dhaka city and high-rise buildings. As for occupation and education level, the majority of them reported as university students and beneficiaries of tertiary level education, respectively.

Respondents from the age group 26–35 years reported significantly higher number of noise-related health problems (B = 0.54, 95% CI: 0.17; 0.91), whereas respondents from the age group 36–45 years reported significantly lower number of noise-related health problem (B = −0.91, 95% CI: −1.55; −0.27) when compared with the youngest age group (18–25 years). As for the effects gender and marital status, male and married respondents reported significantly fewer health problems than female and unmarried ones. Individuals living with their families showed significantly less noise-related health problems (B = −1.33, 95% CI: −1.71; −0.95) than individuals living alone. Respondents living outside Dhaka city or in mixed-use buildings, university students and beneficiaries of tertiary level education reported more noise-related problems than the respondents living in Dhaka city, high-rise buildings, from business as occupation, and having lower than tertiary level education. 

### 3.2. Periods of Extreme Noise Pollution in the Locality

Table 2 shows the perceived extreme noise pollution time in the locality. The majority of the participants of the survey reported noisy environment during school closing hours (11 am to 2 pm) and office morning hours (9 am to 11 am). They also had noisy environment during school morning hours and office closing time.

### 3.3. Source of Information on Noise Levels

Table 3 summarizes the most frequently used sources of noise pollution-related information. The majority of the participants of the survey reported that they mainly used the internet (35%), social media (25%), electronic media such as TV and Radio (22%), and university (18%) as a source of information on noise pollution. Few respondents (<5%) had this information from the authorities.

### 3.4. Noise Pollution in the Locality

As shown in Table 4 that the majority of the respondents (91%) reported noisy environments in the locality with noise generated by different types of sources, where most of them reported two types (34%), followed by more than two types (33%), and at least one type (24%) of noise pollution source. The majority of the respondents reported road vehicles (38%) as a major source of noise pollution in their locality, followed by construction activities (24%) and noise generated by the neighbors (23%). The most noisy time was identified as daytime by 47% of the respondents and as nighttime by 36% of them. 56% of respondents perceived less noise pollution during the COVID-19 lockdown period.

Individuals that reported no noise pollution in their locality showed significantly less noise-related health problems (B = −1.02, 95% CI: −1.64; −0.41). In the case of major sources of noise pollution, respondents that have reported construction activities as a major source of noise pollution showed significantly fewer noise-related health problems than the individuals who reported road vehicles as a major source of noise pollution. However, respondents who were exposed to noise generated by industry reported significantly higher level of noise-related health problems (B = 0.99, 95% CI: 0.38; 1.59) than respondents who were exposed to noise generated by road vehicles. Respondents having noisy environment at nighttime reported a significantly higher number of noise-related health problems (B = 0.59, 95% CI: 0.03; 1.16) than respondents who were exposed noise environment at daytime, whereas respondents who had no or little noisy environment during the whole day and night reported significantly fewer health problems (B = −0.91, 95% CI: −1.53; −0.28), compared to the respondents who had the noisiest environment during the daytime. Individuals who faced noise pollution even in the COVID-19 lockdown period, reported significantly more noise-related health problems (B = 0.29, 95% CI: 0.68; 1.82) than individuals who faced as usual noise pollution. 

The data shown in Table 5 demonstrate that the majority of the study population (48%) did not discuss noise pollution with their neighbors, whereas 39% of respondents reported being highly sensitive to noise pollution. 70% of the respondents did not renovate their house to reduce noise pollution, and 36% of the respondents also did not agree to pay an annual fee to reduce the noise pollution. However, 36% of respondents were willing to pay an annual fee for that purpose. Moreover, 66% of the respondents reported that they would prefer a living place free from noise pollution even with high living costs. 

Respondents who had not discussed noise pollution with the neighbors reported significantly less health problems than those that had discussed that question. Significantly more health problems were also reported by the respondents who answered that they might make a renovation of their house to reduce noise pollution than by the respondents who already made such a renovation. Similarly, respondents who were confused to pay an annual fee to reduce noise pollution reported significantly more health problems (B = 0.81, 95% CI: 0.42; 1.20) than who agreed to pay that annual fee. Individuals who had not preferred living places free from noise pollution reported significantly fewer health problems than those who showed such a preference (B = −0.95, 95% CI: −1.28; −0.63). 

## 4. Discussion

We examined the relationship between self-reported noise-related health problems and socio-demographic data, and subjective noise pollution in the location. We observed that participants’ age group, gender, marital status, living/not living with family, location, type of residence, employment, and level of education were all significant predictors of noise-related health problems. Earlier research established a link between socio-demographic status and perceived noise exposure [11,12,25,26]. Additionally, several studies assessed self-reported noise-related health status using socio-demographic data [1,13,21]. Our findings revealed that younger individuals reported more noise-related health problems, similarly as in another study that found that younger persons may be more likely to be impacted by noise [12]. Additionally, one study found a substantial correlation between younger people’s subjective noise estimation and objective noise measurements [11]. These younger individuals may be more annoyed by noise than older adults [12]. On the other hand, other research suggests elderly adults had a greater prevalence of noise-related health problems [27,28]. 

This study reported that females suffered from noise-related distress at a greater rate than males, which is in concordance with the results of a previous study that found that females were more impacted by religious function-related noise [27]. The authors of that study showed, however, that noise from various sources such as loudspeakers, vehicles, and neighborhoods had a similar effect on males and females. We examined that an individual’s low socio-economic status was associated with noise-related health problems, which is in concordance with the results of previous research [28,29]. A mixed-use building may feature noise from various sources (market, flat, small business), thereby increasing the respondents’ noise exposure. We also found that family could play important role to reduce noise-related health problems. Both married respondents and respondents living with their families reported less noise-related health problems. Students at the university demonstrated noise-related distress. Many university students in Bangladesh live alone, without family members, and in substandard housing, which may increase their noise exposure. Furthermore, another study found high noise pollution at major schools and colleges in a small urban area of Bangladesh [19].

We noticed that people in Bangladesh were most exposed to noise during school and office hours when there were a lot of movement of vehicles and people. One qualitative survey also reported similar results: people complained about loud noise from vehicles and people during school hours in Bangladesh [16]. Internet and social media have grown immensely popular in Bangladesh. We also found a similar pattern in the case of other noise pollution-related information sources. The authority should consider developing a plan to raise public awareness about noise pollution using various channels. 

Our research found that the primary cause of noise pollution are road vehicles, which is in concordance with findings of previous research [3,28,29]. In Bangladesh, vehicles’ loud horns are typical even though prohibited [16]. On the other hand, industrial noise pollution had a more significant detrimental effect on the study population. People in Bangladesh were exposed to less noise during the COVID-19 lockdown period, during which people’s movements were restricted [30,31,32,33]. Nevertheless, several individuals reported exposure to noise throughout this period of restrictions. Additionally, they (individuals exposed to noise also during the COVID-19 lockdown period) were shown to be more impacted by noise pollution than those not exposed to noise pollution. This study established that noise has a detrimental effect on one’s health. Individuals who were exposed to less noise endured fewer noise-related health problems. They suffered more when their exposure to noise was high at night. 

Additionally, this study assessed views of the respondents regarding noise pollution. Numerous people were sensitive to noise pollution. A study investigating French citizens documented a link between noise sensitivity and health status [1]. This study found the link higher in highly sensitive (to noise) men. They also observed the significant association between noise sensitivity and fair/poor self-reported health survey in women.

A holistic strategy must be considered to mitigate noise pollution. The Department of Environment of Bangladesh has several guidelines and initiatives to reduce noise pollution [34]. However, the policy needs to be updated. Proper implementation of the policy is also required. A collaborative strategy should be adopted by the authorities to ensure proper knowledge distribution, a positive attitude towards efforts to minimize noise pollution, and preventive activities. Along with periodic noise pollution surveillance, governments must organize campaigns, social mobilization, and communication to educate and train communities on how to combat this serious public health issue. Considering the COVID-19 pandemic, online initiatives using web-based and mobile applications may be effective. Television and social media can also be utilized to educate the public about community-level noise pollution management strategies. Social media has grown in importance as a source of information for the general public in Bangladesh [35]. However, all of these approaches must consider literacy and of their recipients when they are designed and implemented [36]. Public health and disaster management authorities may use these platforms to communicate noise pollution information. Personnel and other important stakeholders must be equipped and trained to mitigate this significant public health concern. The authorities, researchers, companies, and communities must work together to create effective noise pollution mitigation methods. The authorities should impose a higher fine on those liable for noise. Additionally, they might investigate novel solutions such as a green belt to reduce noise pollution [26,37,38]. 

There are various limitations to the current study that should be considered when interpreting the findings. To begin, it’s worth noting that we relied on self-reported health assessments among Bangladesh’s adult population, which is not equal to a clinical diagnosis. As a result, the conclusions of the study should be interpreted carefully. Second, we used non-probability sampling techniques (where respondents were selected conveniently) using an online questionnaire survey which may include certain biases. For instance, respondents may consider socially acceptable replies even though the survey pattern is anonymous. Additionally, all respondents required access to the internet in order to participate in the survey. Third, our study assessed only perceived noise pollution, which may not be associated with the objective noise level. Nonetheless, this exploratory study may give critical information to the Bangladeshi authorities, as well as aid other impacted communities in developing noise pollution reduction initiatives. 

## 5. Conclusions

This baseline study is the first in Bangladesh to examine the association between perceived noise pollution and adult populations’ self-reported health status. As for socio-demographic characteristics, we found that age group, gender, marital status, living/not living with family, location, type of residence, employment, and level of education were found significant factors to influence noise-related health problems. The data indicate that females were more likely to have noise-related health concerns. Additionally, younger people may be more exposed to noise. The findings suggest that low socio-economic level may be a factor in the noise-related health problems. Our findings also found that types of noise pollution, source of noise pollution, and period of noise pollution are significant predictors of noise-related health problems. Nonetheless, further research is required to gain more information about these questions. 

## Figures and Tables

**Table 1:** Overall noise-related health problems of the respondents shown in relation to their socio-demographic profiles (*n* = 1386).

		Overall Noise-Related Health Problems		
Features	*n* (%)	^d^R^2^	B^#^ (95% CI)	*p*-Value
1. Age (Years)				
− 18–25	909 (65.58)		Reference	
− 26–35	328 (23.67)		0.54 (0.17; 0.91) **	0.004
− 36–45	89 (6.42)	0.013	−0.91 (−1.55; −0.27) **	0.005
− >45	60 (4.33)		0.74 (−0.03; 1.51)	0.059
2. Gender				
− Female	776 (55.99)		Reference	
− Male	610 (44.01)	0.008	−0.59 (−0.90; −0.27) ***	0.000
3. Marital status				
− Married	264 (19.05)		Reference	
− Unmarried	1122 (80.95)	0.006	0.60 (0.20; 0.99) **	0.003
4. Living with family				
− No	280 (20.20)		Reference	
− Yes	1106 (79.80)	0.032	−1.33 (−1.71; −0.95) ***	0.000
5. Location				
− Dhaka	833 (60.10)		Reference	
− Outside Dhaka	553 (39.90)	0.028	1.03 (0.71; 1.34) ***	0.000
6. Residence				
− High-rise building ^a^	731 (52.74)		Reference	
− Low-rise building ^b^	355 (25.61)	0.041	−0.04 (−40; 0.33)	0.842
− Mixed-use building ^c^	226 (16.31)		1.48 (1.05; 1.92) ***	0.000
− Other	74 (5.34)		1.44 (0.75; 2.14) ***	0.000
7. Occupation				
− Business	89 (6.42)		Reference	
− Employed	198 (14.29)		0.64 (−0.09; 1.37)	0.090
− Unemployed	199 (14.36)	0.009	0.63 (−10; 1.37)	0.092
− University Students	900 (64.94)		1.08 (0.44; 1.73) ***	0.000
8. Education level				
− <Tertiary	473 (34.13)	0.018	Reference	
− Tertiary	913 (65.87)		1.10 (0.78; 1.43) ***	0.000

B^#^ = Beta coefficient; ** *p* < 0.01; *** *p* < 0.001. ^a^ High-rise building = more than 5 storey, ^b^ Low-rise building = less than or equal 5 storey, ^c^ Mixed-use building = accommodation, shops, market etc. together. ^d^R^2^ = coefficient of determination.

**Table 2:** Respondents that have indicated a specific time interval as a period of extreme noise pollution in their locality (*n* = 1386).

Time	*n*	*%*
School morning hours (8 am to 9 am)	134	9.67
Office morning hours (9 am to 11 am)	450	32.47
School closing hours (11 am to 2 pm)	466	33.62
Lunch hour (2 pm to 3 pm)	68	4.91
Tutorial time (3 pm to 5 pm)	66	4.76
Office closing time (5 pm to 7 pm)	137	9.88
Other time (7 pm to 10 pm)	65	4.69

**Table 3:** Media used as sources of information about noise pollution (*n* = 1386).

Time	*n*	*%*
Electronic media (TV, Radio)	303	21.86
Internet	459	33.12
National and local authorities	15	1.08
Other People	67	4.83
Printing media	50	3.61
Social media	312	22.51
University	180	12.99

**Table 4:** Association between perceived noise pollution in the locality and overall noise-related health problems (*n* = 1386).

		Overall Noise-Related Health Problems		
Features	*n* (%)	^d^R^2^	B^#^ (95% CI)	*p*-Value
1. Type of noise pollution source				
− 1 type	334 (24.10)		Reference	
− 2 types	473 (34.13)	0.013	0.33 (−0.08; 0.74)	0.113
− >2 types	458 (33.04)		−0.09 (−0.51; 0.32)	0.640
− None	121 (8.73)		−1.02 (−1.64; −0.41) **	0.001
2. Major source of noise pollution				
− Road vehicles	526 (37.95)		Reference	
− Construction activities	328 (23.67)		−0.51(−0.91; −0.10) *	0.014
− Industry	109 (7.86)	0.015	0.99 (0.38; 1.59) **	0.001
− Neighbors	316 (22.80)		−0.37 (−0.78; 0.04)	0.076
− Miscellaneous	107 (7.72)		0.063 (−0.54; 0.67)	0.840
3. Most noisy time				
− Daytime	659 (47.55)		Reference	
− Night-time	125 (9.02)	0.011	0.59 (0.03; 1.16) *	0.038
− Noisy day and night	504 (36.36)		0.29 (−0.05; 0.63)	0.091
− Not that much noisy day and night	98 (7.07)		−0.91 (−1.53; −0.28) **	0.004
4. Noise pollution during COVID-19 lockdown				
− As usual	481 (34.70)		Reference	
− Less than before	778 (56.13)	0.017	−0.16 (−0.50; 0.17)	0.338
− More than before	127 (9.16)		0.29 (0.68; 1.82) ***	0.000

B^#^ = Beta coefficient; * *p* < 0.05; ** *p* < 0.01; *** *p* < 0.001. ^d^R^2^ = coefficient of determination.

**Table 5:** Association between individual views concerning noise pollution and overall noise-related health problems (*n* = 1386).

		Overall Noise-Related Health Problem		
Features	*n* (%)	^d^R^2^	B^#^ (95% CI)	*p*-Value
1. Discussion about noise pollution with neighbors				
− Yes	377 (27.20)		Reference	
− Sometimes	344 (24.82)	0.024	−0.34 (−0.76; 0.09)	0.122
− No	665 (47.98)		−1.08 (−1.45; 0.71) ***	0.000
2. Sensitive to noise pollution				
− High	538 (38.82)		Reference	
− Moderate	694 (50.07)	0.003	−0.25 (−0.18; 0.88)	0.140
− Low	154 (11.11)		0.35 (−0.58; 0.08)	0.194
3. House renovation to reduce noise pollution				
− Yes	212 (15.30)		Reference	
− Maybe	201 (14.50)	0.018	0.88 (0.31; 1.44) **	0.002
− No	973 (70.20)		−0.32 (−0.76; 0.11)	0.148
4. Willing to pay annual fee to reduce noise pollution				
− Yes	495 (35.71)		Reference	
− Maybe	396 (28.57)	0.014	0.81 (0.42; 1.20) ***	0.000
− No	495 (35.71)		0.00 (−0.36; 0.37)	0.996
5. Prefer living place free from noise pollution even with high living cost				
− Yes	913 (65.87)		Reference	
− No	473 (34.13)	0.023	−0.95 (−1.28; −0.63) ***	0.000

B^#^ = Beta coefficient; ** *p* < 0.01; *** *p* < 0.001. ^d^R^2^ = coefficient of determination.

## Data Availability

The data presented in this study are available on request from the corresponding author.
